# Discovery of Novel Glucagon Receptor Antagonists Using Combined Pharmacophore Modeling and Docking

**Published:** 2018

**Authors:** Fataneh Jafari, Amin Nowroozi, Mohsen Shahlaei

**Affiliations:** a *Pharmaceutical Sciences Research Center, Kermanshah University of Medical Sciences, Kermanshah, Iran. *; b *Nano Drug Delivery Research Center, School of Pharmacy, Kermanshah University of Medical Sciences, Kermanshah, Iran. *; c *Medical Biology Research Center, Kermanshah University of Medical Sciences, Kermanshah, Iran.*

**Keywords:** Glucagon Receptor Antagonist, Ligand-Based Pharmacophore Modeling, Computer Aided Drug Design, Docking, Virtual Screening

## Abstract

Glucagon and the glucagon receptor are most important molecules control over blood glucose concentrations. These two molecules are very important to studies of type 2 diabetic patients. In literature, several classes of small molecule antagonists of the human glucagon receptor have been reported. Glucagon receptor antagonist could decrease hepatic glucose output and improve glucose control in diabetic patients. In this research, to identify novel and diverse leads for use in potent glucagon receptor antagonist design, a ligand-based pharmacophore modeling, was developed using the best conformations of training set compounds. The best five features pharmacophore model, called Hypo1, includes, hydrogen bond acceptors, two hydrophobic, and positive ionizable features, which has the highest correlation coefficient (0.805), cost difference (64.38), low *RMS* (2.148), as well as it shows a high goodness of fit and enrichment factor. The generated pharmacophore model has been validated by using a series of similar structures with varying affinities for the glucagon receptor. Then, the developed model has been applied as a search query in different database searching with the main objective of finding novel molecules which have the potential to be be modified into novel lead compounds. As a result, some hit molecules were introduced as final candidates by employing virtual screening and molecular docking procedure simultaneously. The results from pharmacophore modeling and molecular docking are complementary to each other and could serve as a useful way for the discovery of potent small molecules as glucagon receptor antagonist.

## Introduction

G-Protein coupled receptors (GPCRs), a superfamily of membrane proteins, are important targets for drug and in pharmaceutical research too (1). Based on recent reports, GPCRs account for 40–50% of all current drug targets (2-4). These membrane receptors also are targets of 26 out of the 100 top-selling drugs in market (2-4). It must be noted that the GPCR superfamily is the largest gene family in the human genome as GPCRs is inclusive about 5% of human genes (5, 6), Based on what is mentioned above, developing new lead compounds for human GPCRs is very important and attractive. Due to technical problems in determine the 3D structure of these membrane receptors at the atomic level, traditional computer-aided drug design for GPCRs heavily rely on ligand-based modeling approaches (7, 8). 

Glucagon is key hormone that acts as the major counter-regulatory hormone to insulin in the control of glucose homeostasis (9, 10). When the glucagon is bound to the G-protein coupled glucagon receptor, hepatic glycogenolysis and gluconeogenesis are stimulated. Studies propose that molecules with glucagon receptor antagonism property could decrease hepatic glucose output and improve glucose control in diabetic patients. It has also been shown that a small molecule glucagon receptor antagonist can effectively block the glucose response to a glucagon challenge in healthy humans (13). Therefore, glucagon receptor antagonism is being followed as a hopeful approach to treat type 2 diabetes. Given the role of glucagon in the development and maintenance of diabetes in both humans and animals, inhibition of the glucagon signaling pathway may represent a potential new approach for diabetes treatment (14, 15). 

Computer-aided drug design (CADD) is a very useful approach in logical drug design and development to reduce the time and cost for identification, characterization and structure-optimization for novel drug candidates (19-22). CADD can also be useful for logical plan of prodrugs. Prodrugs are generally designed to increase the specificity or bioavailability of the main drug molecules (23-25). In the first report by Paul Ehrlich in early 1900, a “pharmacophore” is defined as a “molecular framework that takes (phoros) the essential features responsible for a drug’s (pharmacon) biological activity” (26). With the increase in generation and in knowledge of the three-dimensional structure of molecules, this notion protected to also set the required of arrangement of essential molecular “features”, e.g. steric, and electrostatic characteristics or hydrogen-bonding abilities (27). The pharmacophore model can be derived either from a receptor binding site (direct method), or from a set of active ligands (indirect method) (28).

Ligand-based pharmacophore modeling has become a key computational strategy for simplify drug discovery in the absence of a macromolecular target structure (32, 33). It is often performed by extracting ordinary chemical features from 3D structures of a set of known ligands envoy of necessary interactions between the ligands and a special macromolecular target. In general, pharmacophore generation from multiple ligands (generally called training set compounds) involves two main phases: creating the conformational space for each ligand in the training set to show conformational flexibility of ligands, and equaling the multiple ligands in the training set and specifying the necessary usual chemical features to make pharmacophore models (34). Ligand-based drug design is an indirect method to simplify the development of pharmacologically active compounds by studying molecules that interact with the biological target of interest (35). Ligand-based drug design methods are beneficial in the lack of an experimental 3D structure (27, 36-38). Due to the loss of an experimental structure, the known ligand molecules that bind to the drug target are studied to find out the structural and physico-chemical properties of the ligands that correlate with the demanded pharmacological activity of those ligands (35). In this study, several structural from Maybridge database were filtered by using the drug-like ADMET properties, such as Lipinski′s rule of five (39). Afterward, a qualitative pharmacophore model was developed based on glucagon receptor antagonists that were acquired from the recently published research (40, 41) and was successfully used in the further screening of the database compounds. The lead molecules were chosen based on their best fit values and then subjected to docking analyses to refine the list of retrieved hits. This research has resulted in to introduce a set of hit molecules as possible candidates for the designing of potential glucagon receptor antagonists. 

## Experimental

*Dataset*

A sufficiently large set of molecules with their glucagon receptor antagonist activity data is required for pharmacophore model generation. Using published data in literature (40, 41) the compounds were found to have IC_50_ values identified using the same biological assay conditions. The IC_50_ value is defined as the concentration of a compound needed to inhibit 50% of the glucagon receptor activity. ChemDraw and Chem3D sprograms were applied to drawn 2D structure and conversion into 3D structure. Then, energy minimization procedure was carried out using PM3 approach for each compound. The generated 3D structure was manually investigated to ensure that the chirality of the chiral molecule is correctly prepared and no structure of molecules was duplicated. The molecular structures were optimized using the Polak-Ribiere algorithm until the root mean square gradient was 0.01 kcal mol^-1^. The selection of an appropriate training set is one of the most main steps in pharmacophore modeling procedure. This step is responsible for the quality of the generated pharmacophore hypothesis. The test set, which is not employed in model building step, but in the pharmacophore validation procedure, has equal weight.

Using Kenard and Stone algorithm the data set was split into a training set and a test set. In running of Kennard and Stone algorithm the calculated features matrix were used as input (42). Molecules were further separated into the training (20 compounds), and test (39 compounds) sets.The studied molecules and structural details are reported in Table 1.

**Table 1:** Details of compounds used in this study: (A) Training Set, (B) Test Set

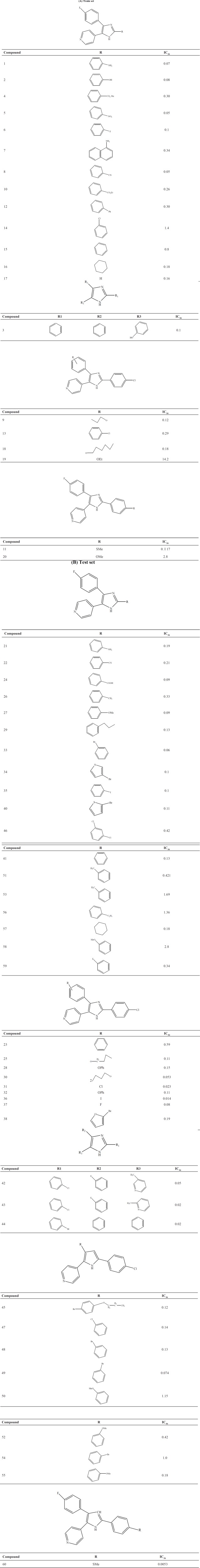

The IC_50_ values of these 59 compounds spanned across a wide range from 0.01µM to 14µM. In this study, 20 out of 59 compounds were chosen as the training set based on the diversity observed in chemical structures and experimental activity values. The remaining 39 compounds were employed in the validation process as the test set. The activity values of the data set were classified into four categories, active (IC_50_ ≤ 0.5 µM, ++++), moderately active ((0.5 ≤ IC_50_ ≤ 5 µM, +++), less active (5 ≤ IC_50_ ≤ 50, ++), and inactive (IC_50_ > 50 µM +), to simplify the results of pharmacophore model building and validation.

*Molecular docking enabled pharmacophore modeling*

Every pharmacophore modeling study that employs the “3D Pharmacophore Generation”or “Common Feature Pharmacophore Generation”protocols of Accelerys Discovery Studio (ADS) conventionally starts with the diverse conformation generation step. The conformations of the selected molecules were generated using the “best conformer generation” method with a cut off value of 20 kcal/ mol from the local energy minimum conformation (23, 43, 44). Pharmacophore model generation was performed using generated conformations of each training set molecule. “Feature Mapping”protocol was applied to identify the chemical features present in all training set molecules. Pharmacophore model generation was performed by choosing chemical features, such as hydrogen bond acceptor (HBA), hydrogen bond donor (HBD), hydrophobic (HYP), and positive ionizable (PI).

Each of the molecules in training set was submitted to the common feature pharmacophore generation procedure. The “common feature pharmacophore generation”protocol implemented in ADS was used to identify and overlay common features shared by a training set. Hypothesis generation run develops 10 possible pharmacophore hypotheses having a different arrangement of above mentioned features and sorts them according to the ranking scores. The Uncertainty value was changed to 2.5 from the default value of 3 as the training set molecules that scarcely spanned the required range of bioactivity (i.e., four orders of magnitude) (45). The Uncertainty value of 1.5 is defined by program as a measured value being 2.5 times higher or 2.5 times lower than the true value.

*Pharmacophore validation*

Commonly developed pharmacophore models are usually employed as 3D queries to search chemical databases to discover new and highly potent lead compounds. The developed pharmacophore models should be statistically significant and able to predict the pIC_50_s of new molecules and retrieve active molecules from the database. The best pharmacophore model was validated employing four techniques, cost analysis, test set prediction, Fischer randomization test, and enrichment factor calculation (E). Common feature pharmacophore generation protocol ranks the 10 generated hypothesis pharmacophore models on their cost values. The weight, error and configuration costs are three components that build the overall cost of a hypothesis. The value of the weight cost increases in a Gaussian form as this function weighs a model′s deviation from the ideal value of two. The error cost value shows the root mean square (*RMS*) difference between the observed and predicted pIC_50_s of the training set molecules. The configuration cost denotes the complexity or the entropy of the conformational space being optimized and is constant for a given data set.

Common feature pharmacophore generation also calculates two additional costs for each hypothesis, fixed cost, and null cost, and also, a cost for every hypothesis namely total cost. The fixed cost is the lowest possible cost denoting a simplest hypothetical model that fits all data completely and the null cost denotes the maximum cost of a pharmacophore with no features and estimates the biological activity to be the average activity of the molecules in training set and the total cost for every hypothesis. A larger difference between the fixed and null costs than that between the fixed and total costs signifies the quality of a pharmacophore model. All of these cost values are reported in bits and a difference of 40–60 bits between the total and null costs suggests a 75–90% chance of representing a true correlation in the data. 20 molecules were used as the test set to validate the developed hypothesis. Fischer randomization (Cat-Scramble) is another technique for pharmacophore model validation. The 95% confidence level was selected in this validation study and 19 random spreadsheets were built. This validation technique checks the correlation between the chemical structures and pIC_50_. This technique produces model employing the same parameters as those employed to generate the original pharmacophore model by shuffling the pIC_50_s of the training set molecules. The fourth way for validating the developed pharmacophore model is based on the E value, which is estimated employing a database containing active and inactive molecules.

*Enrichment factor calculation*

The GH scoring method or Güner-Henry scoring method (46, 47) was employed following external test set validation to evaluate the quality of the developed pharmacophore model. The GH score has been successfully applied to quantify model selectivity precision of hits and the recall of actives from a 1733 (D) molecule dataset consisting of known actives and in-actives.

Of these molecules, 39 compounds (A) are known inhibitors of glucagon antagonists that were selected from literature (30-34) while the other 1694 molecules were from the already prepared chemical dataset. The Güner-Henry scoring method consists of calculating the following parameters: the percent yield of actives in a database (%Y, recall) the percent ratio of actives in the hit list (%A, precision), and the enrichment factor E, and the GH score. The following formula was used to calculate the different parameters 

E = (Ha×D)/ (Ht×A).

The GH score ranges from 0 to 1, where a value of 1 signifies the ideal model.

$\mathrm{\%A}=\frac{\mathrm{Ha}}{A}\times 100$

$\mathrm{\%A}=\frac{\mathrm{Ha}}{\mathrm{Ht}}\times 100$

$E=\frac{\mathrm{Ha}/\mathrm{Ht}}{A/D}$

$\mathrm{GH}=\left(\frac{\mathrm{Ha}(3A+\mathrm{Ht}}{4\mathrm{HtA}}\right)\left(1-\frac{\mathrm{Ht}-\mathrm{Ha}}{D-A}\right)$

*Virtual screening*

Virtual screening, an *in silico* technique for drug design and discovery, has been extensively employed for lead identification in drug discovery process. Virtual screening techniques are generally divided into ligand-based screening and structure-based virtual screening. Pharmacophore-based database searching is considered a type of ligand-based virtual screening, which can be powerfully employed to find novel, potential leads for further development of drug discovery from a released database. A well-validated pharmacophore model includes the chemical features responsible for bioactivities of drug candidates; consequently, it can be applied to carry out a database search. The best pharmacophore model developed was employed as a 3D query in database searching. This virtual screening was carried out to find novel and diverse virtual leads. Leads introduced are appropriate for further drug design and discovery. One of the main advantages of applications of database searching is that the retrieved molecules are typically more easily available for testing than those based on *denovo* design techniques (48). A molecule must be able to map the entire features of the developed hypothesis to be listed as a hit. All screening experiments were carried out employing the Ligand Pharmacophore Mapping protocol with the Best Flexible Search option as available in ADS. Hit molecules from the database searching with less than 0.1 M predicted pIC_50_ values were retained. In addition, hit compounds with good estimated activity were predicted for the drug-likeness using Lipinski′s rule of five (39). A “Lipinski rule of five”said a drug candidate molecule has (i) a molecular weight less than 500; (ii) less than 10 hydrogen bond acceptor groups; (iii) less than 5 hydrogen bond donor groups, and (iv) an octanol/water partition co-efficient (Log P) value less than 5.

*Docking Protocol*

Sixty five compounds that were predicted to be positive in Lipinski drug likeness screening were subjected to molecular docking studies. The crystal structure of glucagon receptor retrieved from the Protein Data Bank (PDB code: 5EE7) was used.

AutoDock Vina is a more recent release of AutoDock program, which uses own scoring function in combination with an Iterated Local Search Global Optimizer. The Vina scoring function is a weighted sum of distance-dependent atom pair interactions, which are defined relative to the surface distance *d*_ij_ (49, 50).

$d_{\mathrm{ij}}=r_{\mathrm{ij}}-(R_{i}+R_{j})$

Here *d*_ij_ is a function of the interatomic distance (*r*_ij_) and the van der Waals radii of the atoms in the pair (*R*_i_ and *R*_j_).

Optimal binding sites were searched in a box of 20 Å in each Cartesian direction. The box had 1.0 Å grid spacing and centered at the allosteric site of the protein. The box was adequately large to include the active site of protein as well as significant regions of the surrounding surface. For this step, default values of parameters were used.

First of all, the internal docking validation was carried out. For this step, the ligand structure was extracted from the Protein Data bank (PDB) file using a plain text editor. After assigning bond orders, missing hydrogen atoms were added and a short minimization (100 steepest descent steps using MM+ force field with a gradient convergence value of 0.05 kcal/mol Å) was performed using HyperChem in order to release any internal strain (51-54). Then, in the AutoDock Tools package, the partial atomic charges were calculated using Gasteiger–Marsili method (55) and after merging non-polar hydrogens, the rotatable bonds were assigned.

For protein, after determining Kollman united atom charges (56) and merging non-polar hydrogens, the rotatable bonds were assigned. 

Applying 2.0 Å clustering tolerance to construct clusters of the closest compounds, the initial coordinates of the ligand were used as the reference structure. Finally, docking results (protein-ligand complexes) were visualized using VMD 1.9.3.

## Results and Discussion

*Pharmacophore modeling*

Before beginning of pharmacophore modeling procedure, a total of 59 glucagon receptor antagonists were gathered from published resources. As mentioned before, of these molecules, 20 were selected to form a training set based on broad coverage of activity range and structural diversity using Kennard and Stone algorithm. The top ten hypotheses were composed of HYP, HYP aliphatic, HBA lipid, HBD, and PI features. Table 2 reports the statistics of the generated pharmacophore hypotheses. The values of the ten hypotheses such as pharmacophore features, root-mean-square deviations (rmsd) correlation (r), cost values, and Fischer confidence levels showed statistical significance (Table 2).

**Table 2:** Results of the top 10 pharmacophore hypotheses generated by the HypoGen algorithm

**Hypothesis**	**Total cost**	**Cost difference ** a	**RMSD**	**Error cost**	**Correlation**	**Features ** b
1	113.989	64.38	2.14865	93.5037	0.805377	HBD, HYP, PI
2	116.752	61.617	2.21863	96.5597	0.790605	HBD, HYP aliphatic, PI
3	120.691	57.678	2.31742	101.041	0.768374	HBD, HYP aliphatic, PI
4	124.388	53.981	2.40129	104.999	0.748323	HBD, HYP aliphatic, PI
5	124.527	53.842	2.26536	2.26536	0.786852	HBA lipid, HBA lipid, HYP aliphatic
6	125.362	53.007	2.4219	105.993	0.74324	HBD, HYP , PI
7	127.274	51.095	2.37537	103.761	0.758553	HBA lipid, HBA lipid, HYP
8	128.58	49.789	2.44362	107.05	0.739536	HBD, HBD
9	134.536	43.833	2.55966	112.855	0.707545	HBA lipid, HYP aliphatic, PI
10	134.67	43.699	2.58868	114.349	0.699263	HBA lipid , HYP aliphatic, PI

Null cost = 178.369; fixed cost = 66.403; configuration cost = 19.818.

a Cost difference = null cost - total cost.

b Abbreviations used for features: hydrogen bond acceptor (HBA), hydrogen bond donor (HBD),hydrophobic (HYP), positive ionizable (PI)

**Figure 1:**
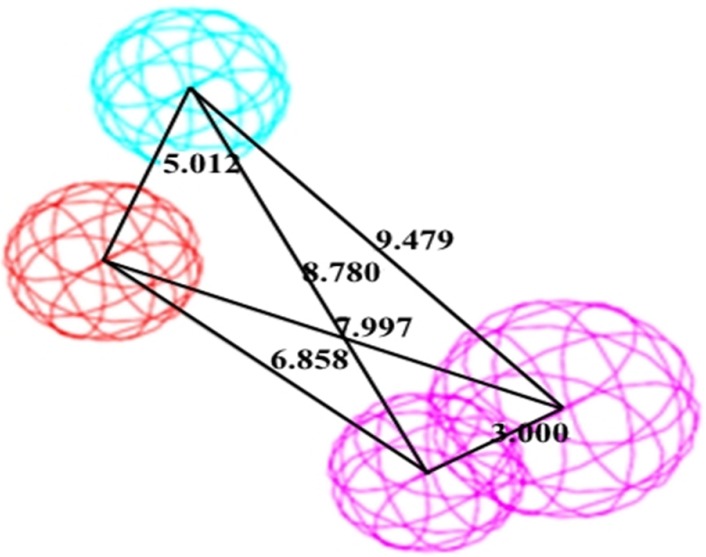
The best pharmacophore model Hypo1 represented with distance constraints. Pharmacophoric features colored as follows: PI (Red), HBD (Violate), hydrophobic (Blue)

A significant pharmacophore model should have a large difference between its total and null cost values. In this work, the best hypothesis, Hypo1, as indicate in Figure 1 and reported in Table 2 is characterized by the lowest total cost value (113.989), the highest cost difference (64.38) and the lowest *RMSD* (2.14865).The developed pharmacophore showed the highest correlation coefficient value of 0.80, highlighting its strong predictive ability. The fixed cost and null cost are 66.403 and 178.369 bits, respectively. The total cost is low and close to the fixed cost, as well as being less and differs greatly from the null cost. The entire evidences indicate that the developed model has good predictive ability. Consequently, Hypo1 was chosen as the best pharmacophore model for further analyses and application.

*Application of developed Pharmacophore model on training set*

**Figure 2:**
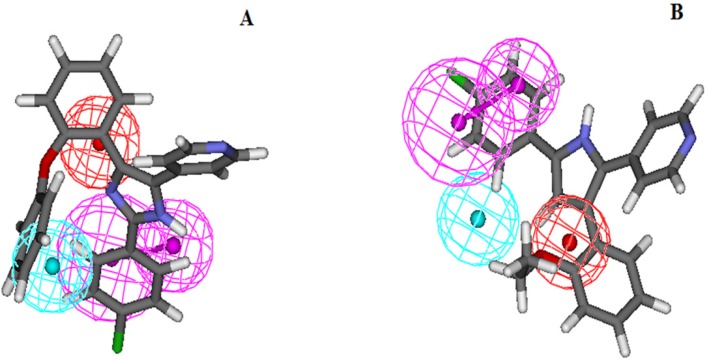
Overlay of most active (A) and least active (B) molecules in the training set upon the best pharmacophore model Hypo1. For details of Pharmacophoric features colors refer to Figure 1

As mentioned above, all of the molecules in the studied dataset set were categorized into four different groups based on their biological activity (IC_50_) values: active (IC_50_ ≤ 0.5µM, ++++), moderately active (0.5 ≤ IC_50_ ≤ 5µM, +++) less active (5 ≤ IC_50_ ≤ 50, ++) and inactive (IC_50_ > 50, +) (Table 3). The activity of each training set molecule was predicted by a fitting procedure based on the best pharmacophore model, Hypo1, and the results are displayed in Table 3. As it can be seen, three of training set molecules were predicted as having different IC50s than their experimental values. It must be noted that all active molecules in the training set were predicted as active glucagon receptor antagonists. One moderately active compound, molecule 19, was underestimated as a less active compound. Also, the estimated activity of two molecules, 16, and 17 were underestimated. Error values depict the ratio between the experimental and estimated activity values. Positive error values are calculated when the predicted activity value is higher than the observed value and a negative value shows the opposite. All of the active compounds listed in Table 3 contained all of the five chemical features in Hypo 1, whereas all of the other compounds mapped four or less pharmacophoric features of hypothesis. Figure. 2A and B depicts the mapping of the most and least active molecules (molecule 13 and 19 respectively) of the training set on Hypo1, respectively.

**Table 3:** Experimental and predicted IC_50_ values of the training set compounds against Hypo1

**Name**	**IC50**		**Fit Value ** a	**Error ** b	**Activity scale ** c	
	**experimental**	**estimate**			**experimental**	**estimate**
1	0.07	0.064	7.36342	-1.08746		
2	0.08	0.068	7.33657	-1.16831		
3	0.1	0.071	7.32137	-1.41017		
4	0.3	0.092	7.2064	-3.24652		
5	0.05	0.096	7.18909	+1.92327		
6	0.12	0.135	7.0406	+1.12802		
7	0.34	0.156	6.97872	-2.17819		
8	0.05	0.163	6.95839	3.27144		
9	0.1	0.164	6.95596	+1.64491		
10	0.26	0.191	6.89303	-1.36742		
11	0.117	0.202	6.8661	+1.72909		
12	0.3	0.232	6.80592	-1.29105		
13	0.29	0.271	6.73877	-1.06922		
14	1.4	0.811	6.263	-1.72595		
15	0.8	0.885	6.22506	+1.1065		
16	0.18	0.8852	6.22506	+4.91778		
17	0.16	0.920137	6.20825	+5.75086		
18	2.68	1.54629	5.98281	-1.73318		
19	14.2	1.81386	5.9135	-7.82861		
20	2.8	2.28485	5.81324	-1.22546		

a Positive value indicates that the predicted IC_50_ is higher than the experimental IC_50_; negative value indicates that the predicted IC_50_ is lower than the experimental IC_50_.

b Fit value indicates how well the features in the pharmacophore map the chemical features in the compound.

c Activity scale: active,

++++ (IC_50_ ≤ 0.5 µM); moderately active,

+++ (0.5 < IC_50_ ≤ 5 µM); less active,

++ (5 < IC_50_ ≤ 50 µM); poor active,

+ (IC_50_ > 50 µM).

*Validation method*

As well as the training set prediction by Hypo1, the predictive ability of the best developed pharmacophore model was tested using additional methods such as cost analysis, prediction of biological activity of test set, Fischer randomization, and E value calculation. Cost analysis is based on the statistical cost values generated during pharmacophore model building phase. A diverse test set was employed to verify if the pharmacophore model predicts the biological IC_50_ of the molecules that are structurally distinct to the training set. The Fischer randomization test was also used to verify that the chosen pharmacophore model was not generated as a result of chance correlation. The E value calculation was built to verify the selectivity of the developed pharmacophore model towards actives molecules rather than in-actives.

*Cost analysis*

The “common feature pharmacophore generation”algorithm generated three cost values during pharmacophore building step to evaluate the quality and reliability of the pharmacophore hypothesis. As described in Method section, the first cost value is the fixed cost value (also called ideal cost) denotes the simplest model that fits the data completely. The second one is the null cost value (no correlation cost) denotes the highest cost of a pharmacophore with no features estimating the activity to be the average IC_50_s of the training set molecules. A statistical significant and predictive pharmacophore hypothesis should have a large difference between these two cost values. Hypo1 was generated with a fixed cost value of 66.403 and a null cost value of 178.369, thus with a difference of 64.38. The third cost is the total cost value estimated for every pharmacophore hyothesis and should be close to the fixed cost value. A large difference between the total and null costs shows a more predictive and statistical meaningful pharmacophore model. Hypo1 scored a total cost value of 113.989, which is closer to the fixed cost, for a cost difference of 134.158 (reported in Table 2).

*Test set prediction*

A set of 39 molecules with structures quite similar to training set and range of IC_50_ values was employed to assess the best developed pharmacophore model, Hypo1. The chemical structures of the test set compounds are provided in Table 1. The “Ligand Pharmacophore Mapping”protocol implemented in ADS with the Best Flexible Search option was applied to map all of the molecules in test data (Table 4). Using this protocol, the activity values were calculated for each molecule in test data. In particular, no compound in the test set was predicted with an error value more than 10, thus not exhibiting more than one order of magnitude between experimental and estimated activities (Table 4). Noticeably, 76.92% (30 molecules) of the test set molecules were predicted within their IC50 scales while the remaining 23.07% (9 molecules) were estimated in different activity scales. From these 9 molecules, 3 active molecules were underestimated as active; 4 moderately active molecules were overestimated as active molecule and 2 less active molecules were overstimated as moderately active and active molecules. All of the less active and inactive compounds were predicted within their activity scales. 

Fit values were calculated using all ten hypotheses and correlated with experimental activities. The best hypothesis, Hypo1, showed a correlation coefficient (R^2^ = 0.805).

**Table 4:** Experimental and predicted IC_50_ data values of the test set compounds against Hypo1

Name	**IC50(µM)**		**Error**	**Fit Value**	**Activity scale**	
	**experimental**	**estimate**			**experimental**	**estimate**
21	0.19	0.059793	-3.177619	7.39545	++++	++++
22	0.21	0.059814	-3.510872	7.3953	++++	++++
23	0.59	0.05992	-9.846429	7.39453	+++	++++
24	0.09	0.060491	-1.487815	7.39041	++++	++++
25	0.11	0.062979	-1.746603	7.3729	++++	++++
26	0.1	0.065565	-1.525216	7.35543	++++	++++
27	0.09	0.065565	-1.372694	7.35543	++++	++++
28	0.15	0.06803	-2.204903	7.3394	++++	++++
29	0.13	0.069603	-1.867725	7.32947	++++	++++
30	0.053	0.072127	+1.360883	7.314	++++	++++
31	0.023	0.072832	+3.166617	7.30978	++++	++++
32	0.027	0.073341	+2.716326	7.30675	++++	++++
33	0.06	0.0735	+1.225003	7.30581	++++	++++
34	0.1	0.073576	-1.359146	7.30537	++++	++++
35	0.1	0.07371	-1.356675	7.30458	++++	++++
36	0.014	0.075042	+5.360157	7.29679	++++	++++
37	0.08	0.075179	-1.064134	7.29601	++++	++++
38	0.19	0.077713	-2.444887	7.28161	++++	++++
39	0.15	0.078717	-1.905558	7.27603	++++	++++
40	0.11	0.095641	-1.150136	7.19146	++++	++++
41	0.13	0.107363	-1.210845	7.14124	++++	++++
42	0.05	0.125811	+2.51622	7.07238	++++	++++
43	0.02	0.160961	+8.04805	6.96538	++++	++++
44	0.04	0.162512	+4.0628	6.96121	++++	++++
45	1.44	0.386826	-3.722604	6.58458	+++	++++
46	0.95	0.429214	-2.213348	6.53943	+++	++++
47	0.14	0.477763	+3.412593	6.49289	++++	++++
48	0.13	0.497664	+3.828185	6.47516	++++	++++
49	0.074	0.5272	+7.124324	6.45012	++++	+++
50	1.15	0.568055	-2.024452	6.41771	+++	+++
51	0.99	0.57235	-1.729711	6.41444	+++	+++
52	0.42	0.577576	+1.375181	6.41049	++++	+++
53	1.69	0.579427	-2.916675	6.4091	+++	+++
54	0.061	0.621097	+10.18192	6.37894	++++	++
55	0.027	0.632999	+23.44441	6.3707	++++	++
56	1.36	0.827474	-1.643556	6.25435	+++	+++
57	0.18	0.8425	+4.680556	6.24653	++++	+++
58	2.8	0.903949	-3.09752	6.21596	+++	+++
59	0.43	1.05557	+2.454814	6.14861	++++	+++
60	0.49	1.1109	+2.267143	6.12642	++++	+++

*Fischer randomization test*

**Figure 3:**
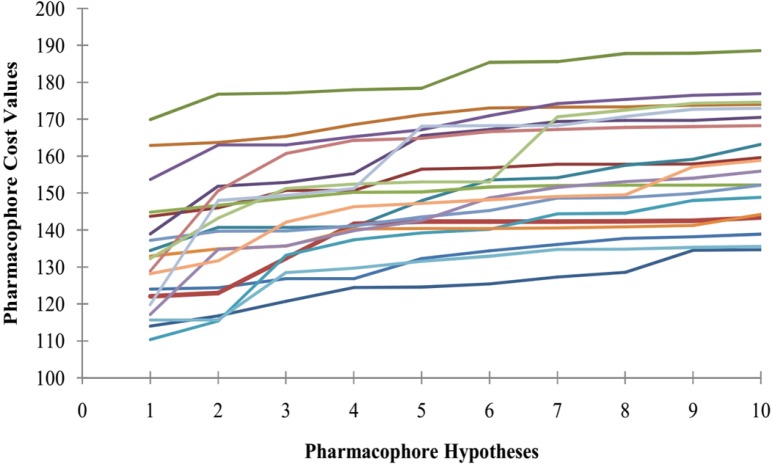
The difference between cost values of developed pharmacophore model and the scrambled models

Furthermore, Fischer randomization test technique was applied to evaluate the statistical robustness of developed pharmacophore model (Hypo1). The third method to validate the robustness of the developed model is based on Fischerʹs randomization technique. The observed biological activities of the training set were shuffled randomly and the resulting training set was used in common feature pharmacophore generation protocol with the parameters selected for the original model building step. Thereby, a set of 19 random tables was generated to reach a 95% confidence level that the best pharmacophore, Hypo1, was not developed by chance. Figure 3 indicates that none of the randomly developed pharmacophore models were generated with better statistical values than Hypo1. The results of Fischer randomization test technique clearly demonstrate that the original hypothesis is far more superior to those of the 19 randomization produced hypotheses, which give confidence on developed pharmacophore model.

*Estimation of Enrichment factor*

The GH score has been effectively used to determine model selectivity (best model) accuracy of hits, and the recall of active molecules from a molecule dataset consisting of known active and inactive molecules. GH scoring methodology has been successfully applied for quantification of model selectivity and coverage of activity space from database mining (57) and for the evaluation of the effectiveness of similarity search in databases containing both structural and biological activity data (58). The GH score contains a coefficient to penalize excessive hit list size and, when evaluating hit lists, is calibrated by weighting the score with respect to the yield and coverage. The GH score ranges from 0, which indicates the null model, to 1, which indicates the ideal model (i.e., containing all of, and only, the active ligands). The GH value is expected to be greater than 0.7 (59). It is considered a relevant metric, as it takes into account both the percent yield of actives in a database (%Y, recall) and the percent ratio of actives in the hit list (%A, precision). The GH scoring formula can be applied to identify best tolerance for an analysis testing different *RMS* tolerance in fixed atomic position. For fixed positions tolerance an optimum GH score can be calculated. This method can also be applied to calculate highest GH score for the activity of a class of compounds clustered in a group. Hence, using the GH score method for each cluster of compounds aimed for particular activity, one can associate ownership for each cluster. 

Generated pharmacophore model was also validated employing which determines whether the best hypothesis can choose active molecules during the virtual screening procedure from a database of 1733 molecules consisting of 39 experimentally determined glucagon receptor antagonists retrieved from the recently published studies. 

Statistics used in this section includes calculation of false positives, false negatives, enrichment factor, and goodness of hit to determine the robustness of the generated hypotheses (47) (reported in Table 5). Not only should the pharmacophore model generated predict the biological activity of the molecules applied for model building, but it should also be skilled for predicting the biological activities of other molecules as active or inactive.

Using the best developed pharmacophore model, Hypo1, 35 molecules (H_t_) were retrieved as hits from the database screening. 

Among these hits, 32 (H_a_) molecules were from the A list of known antagonists. Therefore, the enrichment factor was calculated to be 40.62, indicating that it is 40.62 times more probable to pick an active compound from the database than an inactive one. This value of enrichment factor and a GH score of 0.89 indicated the quality of the model and high efficiency of the screening test.

As it can be seen from this table, selected pharmacophore model is successful in retrieving 90% of the active molecules, 3 inactive molecules (false positives) and predicted 7 active molecules as inactive (false negatives).

**Table 5:** Statistical parameters of GH score validation for Hypo1

**Serial No.**	**Parameters**	**Results**
1	Total number of molecules in database (D)	1733
2	Total number of actives in database (A)	39
3	Total number of hit molecules from the hit database (Ht)	35
4	Total number of active molecules in hit list (Ha)	32
5	% Yield of actives (Ha/Ht) × 100	91.4
6	% Ratio of actives (Ha/A) × 100	82.05
7	Enrichment Factor (EF)	40.62
8	FALSE negative [A-Ha]	7
9	FALSE positives [Ht-Ha]	3
10	Goodness of hit (GH)*	0.89

* [(Ha/4HtA) (3A + Ht) × (1–(Ht –Ha) /(D–A))]

*Virtual Screening and drug-likeness prediction*

In drug design and discovery procedure virtual screening (database searching) is an efficient alternative way to high throughput screening (HTS). The best pharmacophore model, Hypo1, was used as a 3D query to search a chemical database, Maybridge for a total of 174000 compounds. The “Ligand Pharmacophore Mapping protocol”with the Best Flexible Search option was employed to search these databases. Inhibitory activity values were estimated for the compounds obtained from the database screening. A total of 2000 molecules were mapped upon all of the pharmacophoric features present in Hypo1. A total of 100 compounds mapped in previous step scored an estimated activity value less than 0.07 µM and were considered for further studies. 

*Lipinski’s rule of five evaluation*

Drug-likeness properties are one of the key indicators for selecting the molecules for *in-vitro* studies, which includes molecular or physicochemical properties that contribute to favorable Lipinski’s rule of five. The parameters were described in the Lipinski’s rule of ﬁve including logP (the logarithm of octanol/water partition coefficient), number of hydrogen bond donor groups, number of hydrogen bond acceptor groups, and molecular weight. They have been proved to have a correlation with drug absorption. These properties describe the ‘drug-likeness’ and predict a poor oral absorption or permeation when the investigated molecules have more than ﬁve H-bond donors (HBD), 10 H-bond acceptors (HBA) a molecular weight (MW) greater than 500 Da and calculated LogP (cLogP) higher than 5. Compounds breaching more than one of the conditions may have small oral bioavailability. However, among 100 considered compounds, the 66 compounds that are listed in Table 6 did not breach any parameter of Lipinski’s proposed rule, and thus are supposed to have high bioavailability. So, finally 66 molecules were further selected for docking studies.

**Table 6:** Results for the calculated Lipinski’s rule of five

**Molecule code**	**logP**	**MW**	**nON**	**nONHN**
8219	4.89	461.65	7	2
20540	2.37	436.51	8	2
52875	0.36	195.22	4	3
53104	4.56	486.64	6	1
5594	1.34	209.25	4	3
42406	3.38	398.5	6	1
7981	1.2	352.19	7	3
19450	-6.18	367.43	10	3
31628	1.72	408.54	5	1
29669	1.7	496.46	7	3
39647	2.42	465.02	7	2
37962	3. 4	444.63	6	2
39022	3.45	453.55	8	1
24155	1.83	282.44	6	4
22846	3.33	498.63	8	3
38472	4.09	488.59	8	3
21055	5.98	434.63	4	4
39388	3.19	397.47	4	1
12340	-0.73	174.24	4	4
40885	4.52	439.58	6	2
54183	3.17	348.54	4	2
37909	-5.89	387.44	9	2
39337	-0.47	284.22	9	2
53091	0.35	315.33	7	2
38518	2.62	268.31	6	2
52990	-4.29	372.45	7	2
50154	3.44	483.01	7	2
48286	3.6	456.6	8	3
35463	-5.93	296.37	7	2
28032	3.62	456.64	6	3
49622	1.54	264.32	5	1
21648	2.09	248.33	4	1
19696	2.25	462.53	10	5
34378	3.32	379.47	8	1
32291	3	283.78	4	2
821	1.55	237.3	4	3
1792	2.09	248.33	4	1
10093	2	306.41	5	1
1966	0.36	195.22	4	3
18612	5.12	436.98	4	1
43891	1.34	209.25	4	3
20415	3.38	398.5	6	1
970	-0.13	211.22	5	4
32552	-1.42	402.01	5	3
8272	2.38	329.4	5	2
36902	1.26	235..31	4	2
44033	4.89	461.65	7	2
18412	0.3	333.39	7	1
59021	2.04	403.5	7	1
38608	0.63	261.33	6	1
27733	5.01	633.7	10	2
53227	4.8	467.47	5	2
19739	2.73	437.54	8	2
26319	-5.12	319.35	8	2
24809	-0.44	410.5	9	1
26315	-4.31	381.42	8	2
12709	2.21	290.19	4	2
1968	4.75	458.79	4	1
19736	3.74	423.92	7	2
1969	4.63	494.85	5	1
21083	0.84	430.96	8	1
53437	2.56	357.45	6	1
41692	-0.75	319.12	9	4
53365	-6.13	267.33	7	3
47246	3.62	417.57	6	1

*Docking*

In the internal validation step, MK-0893 was docked onto receptor, according to the above docking protocol. After superimposing the experimental and predicted conformations, the *RMSD *were 1.88Å, which is considered as successfully docked (60, 61) and indicating that the parameters set for the AutoDock Vina simulations are reasonable for reproducing the X-ray structures. This result demonstrates that these *in silico* methods are quite robust and suitable for assessing the interaction of such ligands with Glucagon receptor.

The proposed approach was further validated by docking a series of retrieved inhibitors (The 87 hit compounds that were chosen from the pharmacophore filtering studies) reported in Table 6 in the binding site. Docking studies on binding modes are very informative to clarify key structural characteristics and interactions to provide helpful data for suggesting effective glucagon receptor antagonist. To take a snapshot of the activities and binding affinity of the selected compounds, the predicted binding affinity values for each compound are presented in Table 7.

**Table 7:** Predicted activity is theoretical inhibitory activity calculated using Hypo1. Predicted binding affinity of each compound calculated by Autodock Vina

**Compound**	**Predicted Binding Affinity (kcal/mol)**	**Predicted Activity (nM)**	**Compound**	**Predicted Binding Affinity (kcal/mol)**	**Predicted Activity (nM)**
38472	-7.9	0.061804	49622	-6.3	0.05982
26319	-7.7	0.075916	42406	-6.3	0.066033
26315	-7.7	0.077099	32552	-6.3	0.067345
19450	-7.6	0.064345	19739	-6.3	0.075227
39388	-7.5	0.061199	53091	-6.2	0.060509
24155	-7.5	0.06229	53365	-6.2	0.082079
27733	-7.4	0.074549	8272	-6.1	0.067413
39022	-7.2	0.062373	32291	-6	0.059546
37909	-7.1	0.060783	39337	-6	0.060593
41692	-7.1	0.081302	44033	-6	0.069144
7981	-7	0.064607	18412	-6	0.070891
21083	-7	0.079932	53104	-5.9	0.066588
48286	-6.8	0.060237	821	-5.9	0.059928
53437	-6.8	0.079941	40885	-5.8	0.061049
47246	-6.8	0.082623	43891	-5.8	0.066763
29669	-6.8	0.064053	35463	-5.7	0.060168
19696	-6.7	0.059706	8219	-5.7	0.070047
28032	-6.7	0.059844	19736	-5.7	0.07958
39647	-6.7	0.063692	34378	-5.7	0.059621
59021	-6.7	0.073843	38518	-5.6	0.06042
52990	-6.6	0.060312	10093	-5.6	0.059499
21055	-6.6	0.061294	31628	-5.5	0.064185
1968	-6.6	0.078896	37962	-5.5	0.063519
21648	-6.5	0.059809	5594	-5.5	0.066397
50154	-6.5	0.06022	1966	-5.5	0.066588
22846	-6.5	0.062048	970	-5.5	0.066838
20415	-6.5	0.066815	36902	-5.5	0.067675
20540	-6.5	0.067413	38608	-5.5	0.0743
1792	-6.4	0.059809	53227	-5.5	0.074616
54183	-6.4	0.060812	12709	-5.5	0.078052
18612	-6.4	0.066599	52875	-5.4	0.066852
24809	-6.4	0.07648	12340	-5.1	0.061091
1969	-6.3	0.079728			

**Figure 4:**
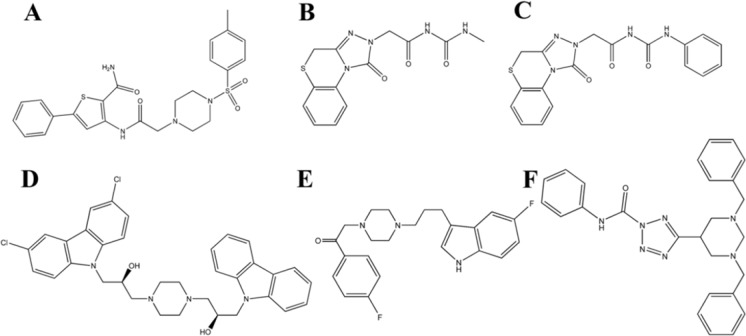
Lead molecules retrieved from the database searching as potent glucagon receptor antagonist. (A) Compound C_38472_ (B) compound C_26319_ (C) compound C_26315_ (D) compound C _19450_ (E) compound C _39388 _and, (F) compound C _24155_

With respect to the obtained results, compound 38472 (maybridge code) was selected for further evaluation. As reported in Table 7, some compounds have more negative estimated binding affinity value than -7.5 which their 2D structures are reported in Figure 4.

On the basis of binding affinity, the order of compounds is: C_38472_> C_26319_> C_26315_> C_19450_> C_39388_> C_24155_

**Figure 5:**
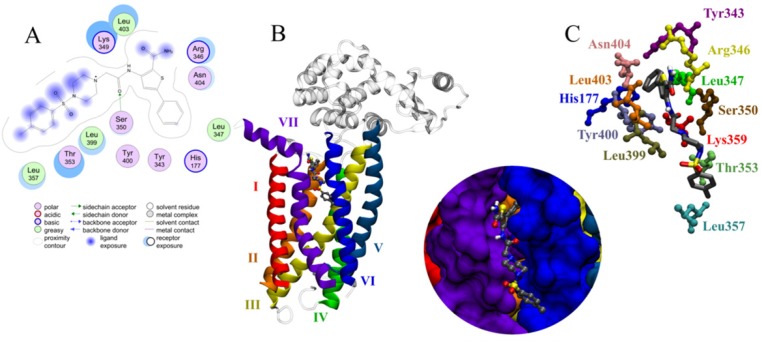
Molecular docking results (A) 2D representation and (B, C) 3D representation of docked orientations of C_38472_ in the binding site of glucagon receptor

As it is obvious, compound C_38472_ interacts more strongly with glucagon receptor site than the other compounds. The binding modes and molecular interactions between compound C_38472_ (Figure 5) (with more binding affinity) and the active site components are discussed below.

As reported in literature (62) the X-ray diffraction studies showed that the residues in outside of the seven transmembrane (7TM) helical bundle in a position between TM6 and TM7, extending into the lipid bilayer, play an important role in the ligand binding.

Compound C_38472_ has a free energy of binding of -7.9 kcal.mol^-1^ and was in contact with the important residues of glucagon receptor such as Ser350, Leu399, Asn404, Thr353 and Lys349 (shown in Figure 5). 

Results of docking study showed that interactions were dominated by the hydrophobicity and aromaticity due to the presence of phenyls, amines, carbonyl and thiophenyl moieties.

The phenyl and thiophenyl rings of compound C38472 is situated in the pocket with high degree of hydrophobic property. This pocket includes the side chains of residues Leu 347, Ser 350, Tyr 400 and Tyr 343.

The carbonyl group between piperazine and amine group of C_38472_ has shown a hydrogen bond interaction with the backbone of SER350.

Binding mode of the compound C_38472_ correlated well with the pharmacophore overlay (Figure 1). 

**Figure 6:**
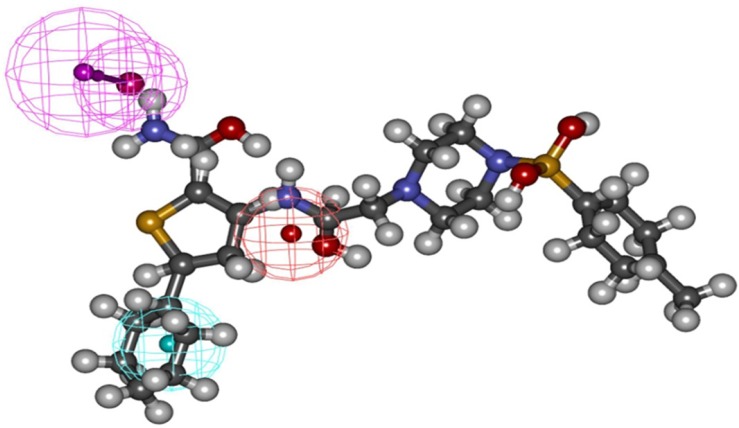
The pharmacophore overlay of the compound C_38472_ on the Hypo1

These findings well corroborates with the best pharmacophore hypothesis where the importance of hydrophobic functionality at the active site has been described by HYDROPHOBIC feature while H-bond interactions at the binding site has been well described by two HBD feature of the pharmacophore (Figure 6).

## Conclusion

Combined computational approaches (pharmacophore modeling and docking approaches) were applied to give insight into the structural basis and inhibition mechanism for a series of glucagon antagonists.

Three-dimensional distances among pharmacophoric features were used as the criteria in the screening process. Since the top 10 pharmacophore models contained the same feature (HBD, HYP, PI, HBA lipid, and HYP aliphatic), first hypothesis (Hypo1) seemed to be the most predictive model with highest rank score. The selected hypothesis was made of HBD, HYP, PI (Figure 1) features with a high correlation value of 0.805 and was validated using 20 molecules assigned as test set compounds. With respect to the obtained results, it appears that the hydrogen bond donor, hydrophobic, and positive ionizable features play an important role, in binding of compounds to the glucagon receptor. This 3D pharmacophore was then further assessed by using it to search 3D databases. Both positive and negative results of this search provided validation of the pharmacophore. The model was further used in database screening to find novel and diverse virtual leads for glucagon receptor antagonist. The retrieved compounds from database searching were further examined at the active site of glucagon receptor where the docking study well corroborates with the pharmacophore model. 

Further, ADME predictions were performed for these compounds. Conclusively, the hits obtained on virtual screening of the database have provided new chemical starting points for design and development of novel glucagon receptor antagonist.
